# Quantification of right ventricular volume in dogs: a comparative study between three-dimensional echocardiography and computed tomography with the reference method magnetic resonance imaging

**DOI:** 10.1186/s12917-014-0242-3

**Published:** 2014-10-12

**Authors:** Anne K Sieslack, Peter Dziallas, Ingo Nolte, Patrick Wefstaedt, Stephan O Hungerbühler

**Affiliations:** Small Animal Clinic, University of Veterinary Medicine Hannover, Foundation, Bünteweg 9, Hannover, D-30559 Germany

**Keywords:** Right ventricular volume, Dog, Three-dimensional echocardiography, Magnetic resonance imaging, Computed tomography

## Abstract

**Background:**

Right ventricular (RV) volume and function are important diagnostic and prognostic factors in dogs with primary or secondary right-sided heart failure. The complex shape of the right ventricle and its retrosternal position make the quantification of its volume difficult. For that reason, only few studies exist, which deal with the determination of RV volume parameters. In human medicine cardiac magnetic resonance imaging (CMRI) is considered to be the reference technique for RV volumetric measurement (Nat Rev Cardiol 7(10):551-563, 2010), but cardiac computed tomography (CCT) and three-dimensional echocardiography (3DE) are other non-invasive methods feasible for RV volume quantification. The purpose of this study was the comparison of 3DE and CCT with CMRI, the gold standard for RV volumetric quantification.

**Results:**

3DE showed significant lower and CCT significant higher right ventricular volumes than CMRI. Both techniques showed very good correlations (R > 0.8) with CMRI for the volumetric parameters end-diastolic volume (EDV) and end-systolic volume (ESV). Ejection fraction (EF) and stroke volume (SV) were not different when considering CCT and CMRI, whereas 3DE showed a significant higher EF and lower SV than CMRI. The 3DE values showed excellent intra-observer variability (<3%) and still acceptable inter-observer variability (<13%).

**Conclusion:**

CCT provides an accurate image quality of the right ventricle with comparable results to the reference method CMRI. CCT overestimates the RV volumes; therefore, it is not an interchangeable method, having the disadvantage as well of needing general anaesthesia. 3DE underestimated the RV-Volumes, which could be explained by the worse image resolution. The excellent correlation between the methods indicates a close relationship between 3DE and CMRI although not directly comparable. 3DE is a promising technique for RV volumetric quantification, but further studies in awake dogs and dogs with heart disease are necessary to evaluate its usefulness in veterinary cardiology.

## Background

The quantification of right ventricular (RV) volume and function is of major clinical relevance regarding morbidity and mortality in patients with pulmonary hypertension, congenital heart disease and congestive heart failure [1–5]. The complex anatomical structures of the right ventricle, such as the crescent shape, the thin wall wrapped around the left ventricle, and the trabeculae of the right ventricular wall, make the evaluation of RV function challenging [6,7]. Consequently, a two-dimensional depiction is not adequate for accurate RV assessment because the right ventricle can only be incompletely visualised in a single view [2,8]. Despite this inaccuracy, two-dimensional echocardiography is the most widely used technique for investigations of the right ventricle in human medicine [5].

The use of three-dimensional (3D) methods promises more accuracy by showing the right ventricle in many planes, enabling a calculation of RV volume independently from model assumptions of ventricular geometry. In many human studies, RV volumetric quantification showed good correlations between 3DE and cardiac magnetic resonance imaging (CMRI) with lower volumes using 3DE [6,9,10]. However, an in vitro study with excised porcine hearts showed good agreement between the 3DE Simpson method and RV volume determination by means of a silicon latex cast [11]. Likewise an in vivo study of 5 dogs, which compared intracavitary balloon measurements and 3DE values, was able to show an accurate assessment of RV volume by means of 3DE [12]. The major advantage of 3DE in veterinary medicine is the unnecessity for general anaesthesia, especially in patients with heart disease. The sectional imaging techniques CMRI and cardiac computed tomography (CCT) provide 3D information of the heart with high spatial resolution [13,14]. Both techniques have proven to be reliable imaging modalities for depicting morphological and anatomical heart structures under physiological and pathological conditions in dogs [15–17]. The need for anaesthesia is the main reason for their reduced use in veterinary medicine. In human medicine, CMRI is regarded as the gold standard for RV volume and function assessment [18] because of its high temporal and spatial resolution and its detailed soft tissue contrast, resulting in high accuracy and reproducibility of the measurements. For this reason, CMRI is considered to be the reference method in many studies investigating RV function [9,10,19,20]. In veterinary medicine, the use of CMRI for RV determination is rare. Only a few studies have been concerned with the right ventricle. In one study it was concluded that technical limitations hamper a good image quality of the moving heart in dogs [21]. However, a more recent study, determining the RV mass in dogs, found accurate results for in vivo CMRI values in comparison to ex vivo measurements [22]. As already mentioned, CCT can be used for cardiac visualisation as well [16]. Nevertheless, in veterinary medicine its use for RV assessment has not been established yet. Considering that veterinary clinics are equipped with a CT scanner more often than an MRI scanner, the evaluation of RV volumetric measurement with CCT is of importance. Therefore, this study focuses on the comparison of RV volumetric quantification by means of 3DE and CCT in comparison with the gold standard CMRI.

## Methods

The study was approved by the Ethical Committee of the Lower Saxony State Office for Consumer Protection and Food Safety (33.9-42502-05-11A133). All study participants were clinic-owned, healthy beagles from the Small Animal Clinic of the University of Veterinary Medicine Hannover, Foundation. The dogs (7 male, 3 female) had a mean age of 6.5 years (±3.26 standard deviation [SD]) and an average body weight of 16.6 kg (±2.08 SD). All dogs included in the study had on the day before the study undergone normal parameters of the physical examination, blood analysis, chest x-ray, electrocardiography, echocardiography and indirect blood pressure (BP) measurement^a^. On the following day, the dogs were anaesthetised for echocardiographic, CMRI and CCT examination using a standard anaesthesia protocol. After induction with levomethadon^b^ (0.2 mg/kg, IV) and diazepam^c^ (0.5 mg/kg, IV), Propofol^d^ (IV) was administered intravenously until endotracheal intubation was possible in a dose up to 0.5 ml/kg. Anaesthesia was maintained with isoflurane^e^ (1.5%) in oxygen. First echocardiography was performed in the anteroom. Due to the lack of an automatic ventilation system in this room, the patient had to be ventilated manually, then CMRI and after that CCT were performed, where the dogs had to be ventilated continuously. The rate of breathing was approximately 12 breaths per minute regardless of being spontaneous, manual or continuous. Throughout the study 0.9% saline was infused at a rate of 3 ml/kg/h. BP was measured during anaesthesia. The duration of anaesthesia from induction till the end of CMRI was 120 minutes.

### 3DE

All echocardiographical examinations were performed using a special heart ultrasound unit^f^ with a V3 matrix-array probe (1.5 – 4 MHz) for 3DE imaging. The probe contains more than 3000 piezocrystals. To record the ECG simultaneously during ultrasound examination, two ECG-electrodes were attached to the skin, one behind the right elbow and the other proximal on the left knee. During examination, the dogs were positioned in left lateral position. For an optimal image quality, a grey scale second-harmonic imaging technique with adjustment of image contrast, frequency, depth and sector size was used. The probe was held on the thorax from left-apical showing the complete long-axis of the two ventricles and their associated atriums. In the following, the plane was optimised for the right ventricle by more cranial probe positioning. To avoid foreshortening of the right ventricle, care had to be taken that the top of the right ventricle was always perpendicular.

**Figure 1:**
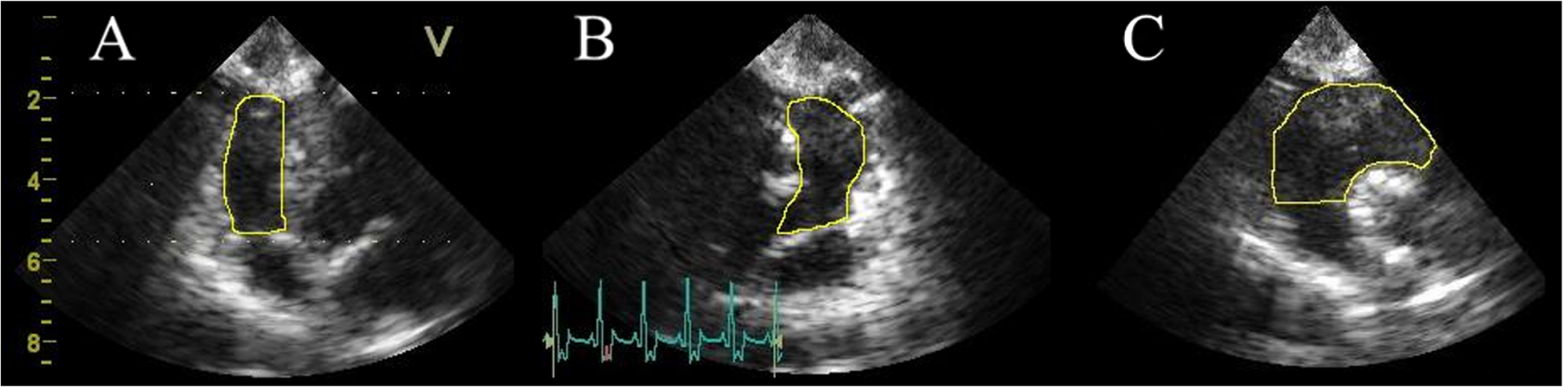
**Three-dimensional echocardiography (3DE) of the right ventricle in an anaesthetised healthy beagle.** The right ventricle is shown in three planes which are 60° to each other: The four-chamber **(A)**, two chamber **(B)** and three chamber view **(C)**. The endocardial contours (yellow line) are manually drawn for better identification and as in all performed methods the papillary muscles and trabeculae were included in the volumes.

In the 4D mode, seven slices were chosen for depicting the right ventricle, which consisted of three axial planes (Figure 1) and four short-axis slices. The first axial plane showed a four-chamber view, the second a two-chamber view (right ventricle and atrium) and the third a three chamber view (inflow- and outflow-tract). The three planes were at angles of approximately 60° to each other. At least, the residual four short-axis slices reached from the apex to the atrioventricular ring. Volume data sets were acquired by single-beat examination with a mean frame rate of 16.8 (min – max = 8.7 to 20) frames/s (fps). During data acquisition care had to be taken that all right ventricular structures were clearly visible in all recorded planes. All echocardiographical examinations were performed by the same experienced cardiologist (S.O.H.).

### Quantification of RV volumes

**Figure 2:**
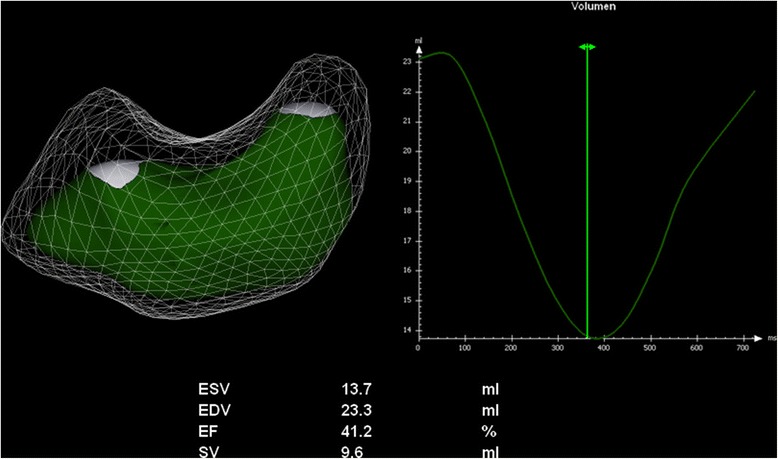
**Results of three-dimensional echocardiography right ventricular function analysis of an anaesthetised healthy beagle.** The model of the RV surface shows the end-diastolic volume (EDV) as transparent volume, whereas the end-systolic volume (ESV) is shown in green. The volume-time curve (VTC) demonstrates the changes of right ventricular volume (y-axis) over time (x-axis). Automatically minimal volume (ESV) and maximal volume (EDV) as well as ejection fraction and stroke volume are calculated from this VTC.

The RV volume 3D data sets were evaluated offline with special software developed for RV quantification^g^. Sagittal, four-chamber and coronal views from one cardiac cycle were loaded into the RV-volume analysis window. After optimal alignment of the RV, markers were placed in the centre of the tricuspid and mitral valve as well as at the LV apex. In the following, initial contours were adjusted in the four-chamber, sagittal and coronal view by manually tracing lines along the RV chamber boundaries. As in the other methods inflow and outflow tract as well as papillary muscles, moderator bands and trabeculae were included in the RV volume [23]. By means of the contour revision function and using the highest sensitivity of contour identification, an entire RV volume set was generated and computed throughout the complete cardiac cycle (Figure 2). Maximum volume was defined as end-diastolic volume (EDV) and minimum volume as end-systolic volume (ESV) as in the other implemented methods. Based on a software integrated physic modelling algorithm, the EDV and ESV were assessed. Stroke volume (SV) was defined as the difference between EDV and ESV and ejection fraction (EF) as the percentage change of these volumes.

### CMR imaging

The CMRI examination was performed with a 3.0 Tesla MRI^h^. Four MRI-compatible ECG electrodes^i^ were stuck on the shaved skin at the left chest wall by positioning the first three at a right angle to each other and the last one slightly caudal of them. In supine position, four surface coils^j^ were placed in an overlapping technique dorsal and ventral on the thorax. To avoid motion artifacts, scans were recorded during end-expiratory breath-holds.

For scanning, a fast field echo (FFE) sequence was used with the following parameters: TE (time to echo) 2 ms; TR (time to repeat) 4 ms, flip angle 40°; matrix 256 × 256 and pixel size 1.2 × 1.2. Image acquisition took place with dedicated scan software^k^. The scans started with compiling survey images of the cardiac region in sagittal, transversal and coronal orientation. From these survey images a short-axis stack was planned, which was adjusted to be parallel to the tricuspid valve and perpendicular to the septum. During several end-expiratory breath-holds, short-axis slices covering the entire ventricle from heart base to apex were acquired with a slice thickness of 4.0 mm and without a slice gap. Each acquired image stack consisted of 18–22 slices, whereas each slice contained 30 heart phases.

**Figure 3:**
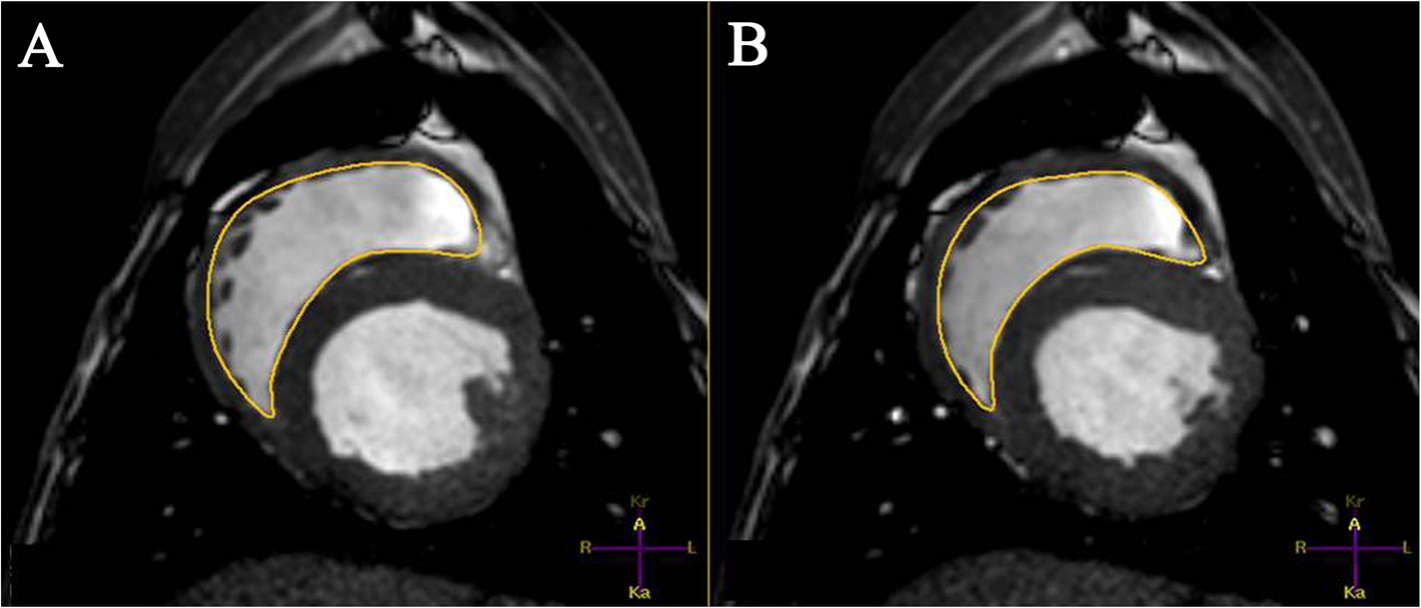
**Cardiac magnetic resonance imaging (CMRI) right ventricular analysis of an anaesthetised healthy beagle.** CMRI-short-axis is shown in **(A)** end-diastolic and **(B)** end-systolic state. The endocardial contours are manually drawn and as in all performed methods the papillary muscles and trabeculae were included in the volumes.

After image acquisition, the evaluation of RV volume was accomplished by means of the disc summation method. Contours were drawn manually along the endocardial boundaries from the apex to the base of the right ventricle in end-diastolic and end-systolic images (Figure 3). The end-diastolic image was defined as the frame with maximum dilatation and the end-systolic image as the frame with maximum contraction. The first slice with a visible RV lumen was identified as the apex. In the basal slice, when the pulmonary valve was visible, contours were drawn up to the junction with the valves [24] because the RV outflow tract should be included in the measurements. Moreover, papillary muscles and trabeculae were attributed to the RV volume, whereas the interventricular septum, epicardial fat, and the pericardium were excluded. For determining EDV and ESV the disk summation method was used [25]. SV and EF were calculated as in 3DE.

### CCT examination

CCT images were obtained by using a 64-detector-row CT^l^ with a gantry rotation time of 400 ms, tube current of 400 mA, tube voltage of 120 kV, detector collimation of 64 × 0.625 mm and a table pitch of 0.20. The dogs were placed in supine position and three ECG electrodes were applied to both forepaws and to the left hind paw for simultaneous ECG-recording during the scans. Scans were also performed during apnea.

At first, survey images from thorax entrance to diaphragm were created in sagittal and dorsal orientation in which the region of interest (ROI), the heart, was adjusted. Additionally, a small ROI was positioned in the ascending aorta for an automated bolus tracking technique [26]. For this purpose, an iodinated contrast medium^m^ (2 ml/kg, IV) was administered using a power injector^n^ into a peripheral vein and the density of Hounsfield units (HU) was measured in the ROI of the aorta. When the threshold of 110 HU and an additional delay of 3.3 sec had been exceeded, the helical scan started automatically.

Retrospectively, the spiral CT datasets were reconstructed in 10% steps throughout the cardiac cycle using a multi-segmental reconstruction algorithm. Thus, 10 heart phases emerged, which were shown as multiplanar reconstructions (MPR) with 0.9 mm slice thickness, 0.45 mm increment, 512 × 512 reconstruction matrix and an individual field of view.

**Figure 4:**
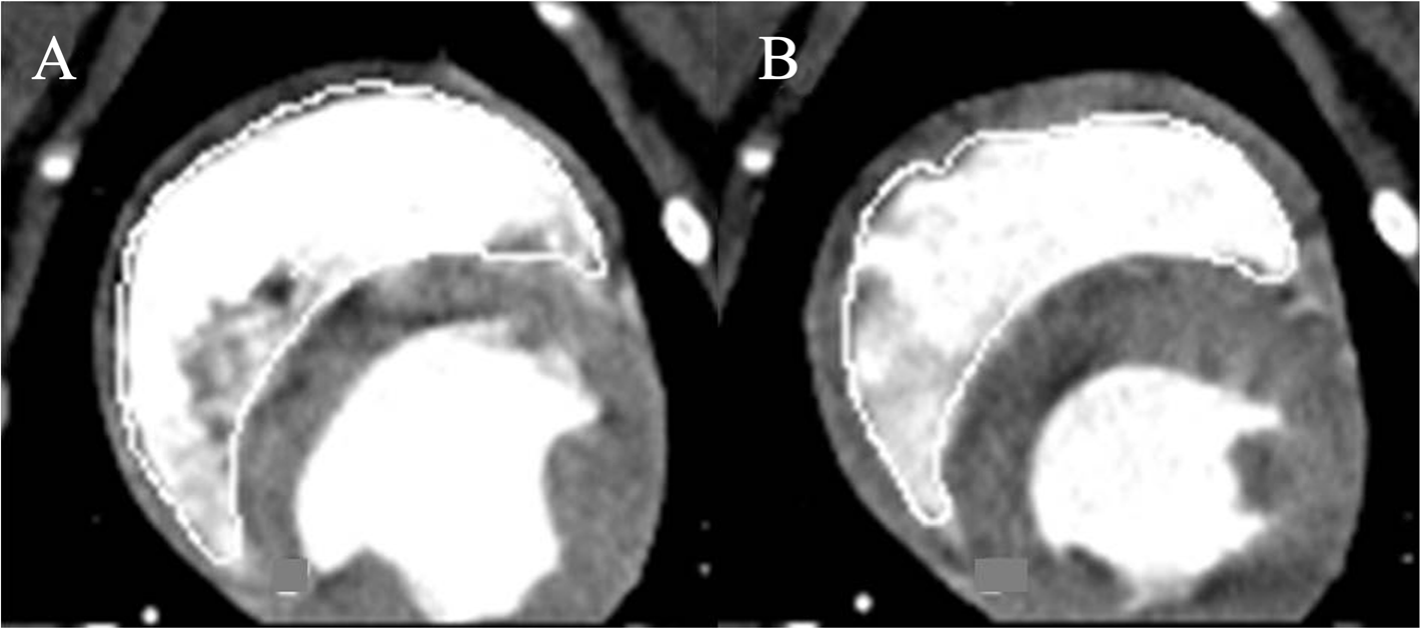
**Midventricular short-axis of the right ventricle in cardiac computed tomography (CCT) of an anaesthetised healthy beagle.** In CCT-short-axis the endocardial border is manually traced in **(A)** end-diastolic and **(B)** end-systolic volume and as in all performed methods the papillary muscles and trabeculae were included in the volumes.

Data analysis was accomplished in the Extended Brilliance Workspace^o^. In order to create short-axis images, the MPRs were loaded in a Cardiac viewer^p^ with multiplanar view modus that showed three planes of the heart: the vertical long-axis, the horizontal long-axis and the short-axis plane. Axes had to be corrected manually. The short-axis reformations were generated in both long-axes planes parallel to the tricuspid valve and perpendicular to the septum. Phases with maximum dilatation and maximum contraction were defined as end-diastole and end-systole (Figure 4). Last of all, short-axis stacks were produced covering the entire right ventricle with 16 slices, a slice thickness of 3.0 mm and an interslice gap of 3.7 – 4.1 mm (min – max). For evaluating the RV volume, the disk summation method was used. For this, the end-diastolic and the end-systolic short-axis stacks were loaded into the LV/RV Analysis software^q^. The same conditions for slice selection were applied as for CMRI. Despite the use of semi-automated border detection, manual correction was necessary.

### Statistical analysis

For statistical tests and graphic presentation special software^r^ was used. Heart rates and values of EDV, ESV, SV and EF were expressed as mean, median and standard deviation. CMRI and CCT data were measured once, whereas 3DE data were calculated as the mean of three heart cycles. To verify normal distribution of data, a Shapiro-Wilk test was performed. In the following, a single factor variance analysis was used to test the null hypothesis that CCT and 3DE programs provide identical results when measuring LV volumes and function compared to CMRI. Multiple pairwise comparisons between the analysing methods were performed to examine whether significant differences occur. A p-value of < 0.05 was considered significant. For each pair of values, limits of agreement and systematic errors were assessed by evaluating the mean difference (bias) and the standard deviation of the differences using the Bland-Altman method. The strength of relation between each analysing technique and CMRI reference values was expressed by linear regression analysis with Spearman correlation coefficients. Correlations were defined as excellent with R ≥ 0.90, very good with R ≥ 0.70 and < 0.90, less good with R ≥ 0.50 and < 0.70 and weak with R < 0.50.

The reproducibility of 3DE data was tested by reevaluating the first analysis by the main investigator (A.K.S.) more than 2 weeks after the first measurement. Relative differences, correlation and significances were calculated for each parameter. To assess the variability, correlation and significances between two observers, the same blinded data were evaluated by a different observer (S.O.H.). The intra-observer and inter-observer variability were expressed as relative difference (percentages), defined as difference of the means between the two measurements, divided by their mean value and multiplied by 100.

## Results

All investigated dogs were clinically healthy with blood values within normal range. They had a regular sinus rhythm and their vertebral heart scales ranged from 10.0 to 11.2 with a mean of 10.5. Their mean systolic BP was 153.3 ± 13.8 mmHg and their mean diastolic BP was 88.1 ± 13.5 mmHg. During echocardiographical examination in four dogs minimal mitral valve regurgitation was found with a reflux smaller than 20% of the left atrium using colour Doppler, and with a broken profile in early systole using continuous-wave Doppler.

In anaesthesia, dogs had a mean systolic BP of 112.3 ± 13.21 mmHg and a mean diastolic BP of 57 ± 14.84 mmHg. The heart rates were not significantly different between the different methods (echocardiographical examination: 96.3 ± 14.33 beats per minute (bpm), CCT: 95.4 ± 11.96 bpm, CMRI 89.8 ± 9.11 bpm).

**Figure 5:**
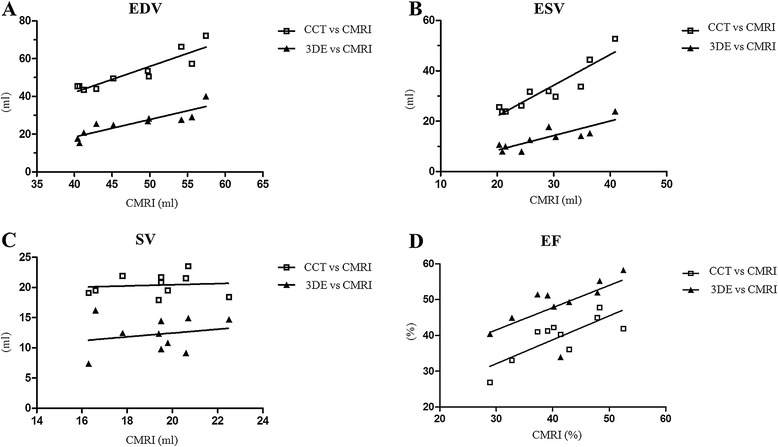
**Scatterplots with correlation analysis of the values of right ventricular function analysis of 10 healthy anaesthetised beagles.** The comparison of cardiac computed tomography (CCT) or three-dimensional echocardiography (3DE) with the reference method cardiac magnetic resonance imaging (CMRI) is presented. Note the approximate linear association between the values of CMRI and CCT, and 3DE of end-diastolic volume (EDV, Figure 5A), end-systolic volume (ESV, Figure 5B) and ejection fraction (EF, Figure 5D). No significant correlation was found for SV (Figure 5C). **A)** EDV comparing CMRI vs CCT and CMRI vs 3DE; **B)** ESV comparing CMRI vs CCT and CMRI vs 3DE; **C)** SV comparing CMRI vs CCT and CMRI vs 3DE; **D)** EF comparing CMRI vs CCT and CMRI vs 3DE.

**Figure 6:**
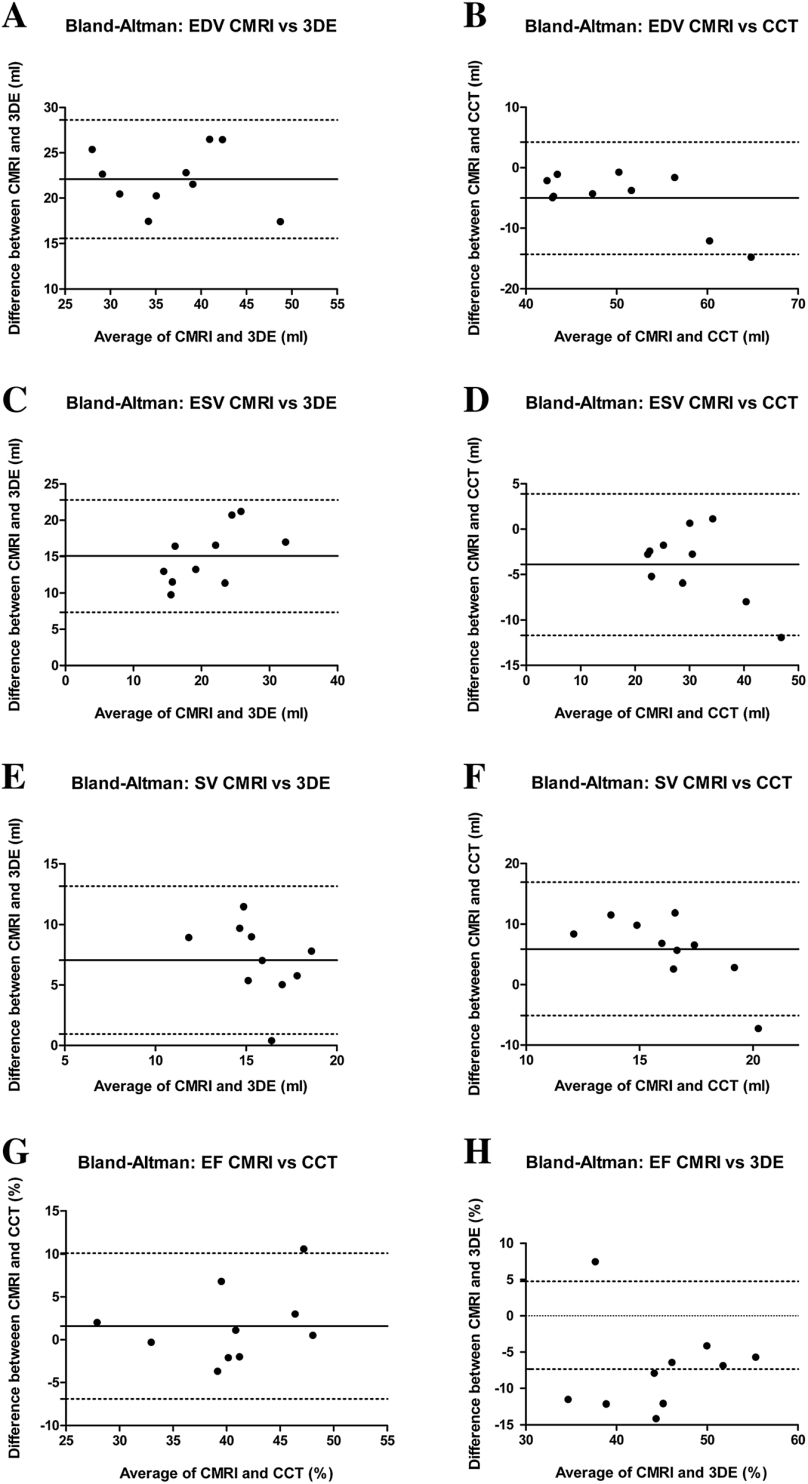
**Bland-Altman plots of the values of right ventricular function analysis of 10 healthy anaesthetised beagles.** The comparison of cardiac computed tomography (CCT) or three-dimensional echocardiography (3DE) with the reference method cardiac magnetic resonance imaging (CMRI) is presented. In each panel, the solid horizontal line represents the mean value of differences (bias) and the dotted lines the limits of agreement (bias ± 1.96 standard deviation). Note that there is an only small bias between CCT and CMRI as opposed to the large bias between 3DE and CMRI (end-diastolic volume = EDV, end-systolic volume = ESV, ejection fraction = EF, stroke volume = SV). **A)** EDV comparing CMRI vs 3DE; **B)** EDV comparing CMRI vs CCT; **C)** ESV comparing CMRI vs 3DE; **D)** ESV comparing CMRI vs CCT; **E)** SV comparing CMRI vs 3DE; **F)** SV comparing CMRI vs CCT; **G)** EF comparing CMRI vs 3DE; **H)** EF comparing CMRI vs CCT.

All examinations were performed without complications. The landmarks RV cavity, RV wall, RV outflow tract (infundibulum, body), tricuspid and pulmonary valve could be found in all image data sets. However, image quality differed between the three image modalities. In the CMRI images all named structures were clearly visible, except tricuspid and pulmonary valves which were more difficult to identify. CCT images were also of good quality, but the RV wall and the valves were slightly more difficult to define. The visualisation of the RV outflow tract was limited in all 3DE data sets. Median, mean ± SD of CMRI, CCT and 3DE examinations are summarised in Table 1, the results of correlation and Bland-Altman analysis in Table 2 and Figures 5 and 6.

**Table 1:** **Right ventricular function variables**

**Variable**	**CMRI**	**CCT**	**3DE**
EDV (ml)	47.73 ± 6.51 (47.46)^a,b^	52.75 ± 9.83 (50.05)^a,c^	25.64 ± 6.87 (26.20)^b,c^
ESV (ml)	28.45 ± 7.13 (27.46)^a,b^	32.35 ± 9.45 (30.70)^a,c^	13.38 ± 4.86 (13.18)^b,c^
SV (ml)	19.27 ± 1.91 (19.50)^b^	20.38 ± 1.78 (20.15)^c^	12.22 ± 2.89 (12.40)^b,c^
EF (%)	41.13 ± 7.21 (40.80)^b^	39.54 ± 6.04 (41.10)^c^	48.47 ± 7.13 (50.25)^b,c^

Mean ± standard deviation values for right ventricular RV function variables (end-diastolic volume = EDV, end-systolic volume = ESV, stroke volume = SV, ejection fraction = EF) obtained with cardiac magnetic resonance imaging (CMRI), cardiac computed tomography (CCT) and three-dimensional echocardiography (3DE) in 10 healthy anaesthetised beagles.

^a, b, c^Significant differences (*p-*value < 0.05); ^a^Significant differences between CMRI and CCT; ^b^Significant differences between CMRI and 3DE; ^c^Significant differences between CCT and 3DE.

**Table 2:** **Statistical evaluation of different methods of right ventricular volume quantification**

		**Group**	**Correlation**		**Bland-Altman analysis**	
**Variable**	**Techniques compared**	**Comparison**	**R**	**P value**	**Bias**	**SD**
	CMRIvs 3DE	<0.0001^a^	0.96	< 0.0001^a^	22.09	3.33
EDV	CMRIvs CCT	0.0084	0.88	0.0009^a^	−5.02	4.73
	CCT vs 3DE	<0.0001^a^	0.80	0.0052	27.11	5.69
	CMRIvs 3DE	<0.0001^a^	0.82	0.0038^a^	15.07	3.94
ESV	CMRIvs CCT	0.0128^a^	0.93	0.0001^a^	−3.9	3.98
	CCT vs 3DE	<0.0001^a^	0.88	0.0008^a^	18.97	5.67
	CMRIvs 3DE	<0.0001^a^	0.20	0.5784	7.05	3.11
SV	CMRIvs CCT	0.1896	0.18	0.6130	−1.11	2.47
	CCT vs 3DE	<0.0001^a^	0.10	0.7763	8.16	3.34
	CMRIvs 3DE	0.0045^a^	0.68	0.0289^a^	−7.34	6.17
EF	CMRIvs CCT	0.2771	0.71	0.0217^a^	1.59	4.35
	CCT vs 3DE	0.0015^a^	0.72	0.0186^a^	−8.93	6.28

Group comparison (GC), correlation coefficients and Bland-Altman analysis (bias and standard deviation [SD]) for values of right ventricular function (end-diastolic volume = EDV, end-systolic volume = ESV, stroke volume = SV, ejection fraction = EF) obtained with cardiac magnetic resonance imaging (CMRI), cardiac computed tomography (CCT) and three-dimensional echocardiography (3DE) in 10 healthy anaesthetised beagles.

^a^Significant differences (*p* < 0.05).

### Values of end-diastolic and end-systolic volume

When comparing with CMRI, 3DE underestimated (bias ± SD: EDV = 22.09 ± 3.33, ESV = 15.07 ± 3.94) the RV volumes significantly (EDV and ESV: p < 0.0001), whereas CCT showed significant (EDV: p = 0.0084, ESV: p = 0.00128) different values with slightly higher volumes (bias ± SD: EDV = −5.02 ± 4.73, ESV = −3.9 ± 3.98), both methods presenting very good to excellent correlations (EDV: CCT: R = 0.88, 3DE: R = 0.96; ESV = CCT: R = 0.93, 3DE: R = 0.82).

### Values of stroke volume

SV was not different (p = 0.1896) between CMRI and CCT (SV [Median ± SD]: CMR = 19.3 ± 1.9 ml; CCT: 20.4 ± 1.8 ml) with little bias (−1.1 ± 2.47 ml) and weak correlation (R = 0.18). When comparing CMRI and 3DE the SV was significantly (p = <0.0001) lower using 3DE (SV: 12.2 ± 2.9) with wide bias (7.05 ± 3.11) and weak correlation (R = 0.20).

### Values of ejection fraction

In comparison to CMRI, EF was significantly (p = 0.045) overestimated (bias ± SD: −7.34 ± 6.17) by 3DE with less good correlation (R = 0.68), whereas using CCT EF was not significantly different (bias ± SD: 1.59 ± 4.35, P = 0.2771) with very good correlation (R = 0.71).

### Reproducibility of 3DE data

For the RV data of 3DE, the relative differences between two observers were found to be 11% for EDV, 13% for ESV, 9% for SV and 1% for EF (Table 3), respectively with little but significant difference only for ESV and very good correlation (R > 0.7) for EDV and ESV, and less good correlations for EF (R = 0.58) and SV (R = 0.26). The relative differences between measurements of the same observer were 2% for EDV, 3% for ESV, 0% for SV and 1% for EF (Table 4), respectively without significant differences and very good correlation for EDV, ESV and EF (R > 0.81) and less good correlation for SV (R = 0.58).

**Table 3:** **Inter-observer variability of three-dimensional echocardiography**

**Variable**	**First observer (ml)**	**Second observer (ml)**	**Relative difference (%)**	**Group comparison (P-Values)**	**Correlation coefficient (R)**
EDV	25.64	28.61	10.94	0.160	0.71
ESV	13.38	15.20	12.74	0.027^a^	0.83
SV	12.22	13.42	9.40	0.556	0.26
EF	48.47	47.99	1.00	0.695	0.58

Comparison of right ventricular function parameters measured with three-dimensional echocardiography by two different observers (end-diastolic volume = EDV, end-systolic volume = ESV, ejection fraction = EF, stroke volume = SV).

^a^Significant differences (*p* < 0.05).

**Table 4:** **Intra-observer variability of three-dimensional echocardiography**

**Variable**	**First measurement (ml)**	**Second measurement (ml)**	**Relative difference (%)**	**Group comparison (P-Values)**	**Correlation coefficient (R)**
EDV	25.64	26.05	1.60	0.695	0.81
ESV	13.38	13.84	3.45	0.625	0.95
SV	12.22	12.22	0.00	0.922	0.58
EF	48.47	48.09	0.80	0.846	0.84

Comparison of right ventricular function parameters measured twice at least two weeks apart with three-dimensional echocardiography by the same observer (end-diastolic volume = EDV, end-systolic volume = ESV, ejection fraction = EF, stroke volume = SV).

## Discussion

In the present study, the right ventricles of 10 Beagle dogs were investigated by means of 3DE and CCT in comparison with the reference method CMRI. The major challenge for determining right ventricular volume is the right ventricle itself. The complex shape with its segmentation in body, inflow and outflow tract, the thin RV wall and the heavy trabeculation make the RV measurement in two-dimensional echocardiography difficult [6]. CMRI is the gold standard for RV volumetric quantification in human medicine [18] and overcomes the limitation of 2D echocardiography by using a three-dimensional volume set, as well as the 3D modalities 3DE and CCT. Although the assessment of left ventricular function using two- and three-dimensional volume estimation is part of various veterinary publications [27–30], there are to the best knowledge of the author no veterinary studies concerning the evaluation of right ventricular function either by means of two-dimensional volume calculation or even three-dimensional volume measurement. Comparing the methods in this study, 3DE would be the most practical in veterinary cardiology, reasoned by the unnecessity for anaesthesia and the inexpensive hardware. The focus of this study was the comparison of 3DE, CCT and CMRI under as comparable conditions as possible; therefore all measurements were performed in anaesthesia. The evaluation of the usefulness of 3DE in awake dogs will be part of further studies. CT scanners in contrast with MRI scanners are nowadays quite common in the veterinary clinic. Therefore, its usefulness for RV volumetric measurement was investigated as well.

The results of the current study showed significant lower RV volumes by using 3DE and significant higher RV volumes by using CCT compared with CMRI with very good to excellent correlations. Differences between the modalities can have various reasons, which are in context with their different image quality, data acquisition and reconstruction, temporal resolution, selection of the last basal slice and the use of contrast agent for CCT examination.

This study was performed in general anaesthesia, whereas human studies have been performed in conscious patients. One can speculate that the negative inotropic effect caused by isofluran [31] results in higher RV volumes (especially ESV) and lower SV and EF the gravest in the last performed modality (CCT) with the longest duration of anaesthesia. Since 3DE was performed as first examination and showed the lowest RV volumes, whereas CCT was performed last with the highest RV volumes, an anaesthesia associated effect cannot be completely ruled out.

Furthermore, different patient positioning could have influenced the results of this study. A lower ventricular filling in supine position with lower stroke volume is described by catheter intervention or thermodilution technique [32–34] in narcotised animals. This is contrary to our results because the lowest RV-volumes exist for 3DE measurements in lateral recumbency, whereas the other modalities with higher volumes were performed in supine position. Therefore, this possible positioning associated effect is probably concealed by the methodical difference caused by the various techniques.

Furthermore, the differing ventilation protocol could have possibly influenced the results in this study because CCT and CMRI were performed in apnoea and 3DE was not. However, we took care not to acquire 3DE volume data sets during breathing. Therefore, this influence could not have been that intense.

In human medicine, the 3DE underestimation of RV volumes compared with CMRI (and CCT) is well known [9]. However, in contrast to our study, the investigations were carried out in conscious human patients with cardiac disease, therefore the results are not directly comparable. In agreement with previous human medical studies [6,9,19,23], we made the experience that several factors influence the measurements of RV cavity in 3DE. One factor is the confounding effect of the apical trabeculae on endocardial tracking [6,9,23]. Furthermore, the worse demarcation of the RV anterior free wall and the RV outflow tract from the surroundings and the indistinct visualisation of the pulmonary valve lead to difficulties in correct contour tracing [19]. Thus, the restricted image quality of 3DE may be the main reason for underestimation of the RV volume. On comparing Figures 1, 3 and 5 it is apparent that the border delineation with CMRI and CCT is far better than with 3DE. Perhaps contrast-enhanced 3D volumetric quantification could be a future aspect for better RV border detection, as shown in human medicine [35].

Another reason causing underestimation of RV volumes could be the low temporal resolution. As the definition of EDV and ESV in all performed methods was based on the maximum and minimum RV volume and not defined according to ECG the measurements were not necessarily carried out at exactly the same time point due to the differing temporal resolution. The lower the temporal resolution, the lower is the probability of detecting the maximum or minimum RV volume. Nevertheless, the definition of EDV and ESV according to ECG is imprecise since, for example, the RV maximum volume exists somewhere in the R wave.

In our study 3DE temporal resolution ranged from 8.7 to 20 fps. Compared with the temporal resolution of CMRI of 30 fps this is rather low. A frame rate of 20 – 40 fps is considered sufficient in left ventricular 3DE volumetric quantification [36,37]. The technical problem of achieving a good temporal as well as spatial resolution is discussed intensively in human cardiology [7,38,39]. In RV volumetric measurement an extremely wide field of view is necessary, which causes low frame rates. Human studies used frame rates of 14 up to 40 fps by using 7 – 8 subvolumes [10,23,40]. The ultrasound machine in the present study is able to generate single-beat volumes and we achieved a mean frame rate of 16.8 fps for RV depiction. This is equivalent to approximately 10 volumes per heart cycle (heart rate = 96 beats per minute), causing a low temporal resolution. Considering CCT, the temporal resolution was comparable to 3DE with 10 frames per heart cycle, whereas CMRI has a temporal resolution of 30 frames per heart cycle. By using 3DE with subvolumes of different heart cycles it is possible to generate higher frame rates, but the spatial resolution is worse. In this study the delineation of the RV endocardium was very difficult, using up to 4 subvolumes without stitching artifacts. In four cases it was not possible to obtain stitching artifact-free images. Consequently, the single-beat mode for RV volume evaluation was used. For precise measurements a high temporal resolution is needed [14]. Therefore, best results are expected with CMRI. Furthermore, the lower temporal resolution of CCT is described as a potential source of error in many publications [41,42]. The same applies to the low temporal resolution of 3DE. Thus, it could be another factor for underestimation of RV volume.

However, the higher RV volumes with CCT explained only by its limited temporal resolution alone is implausible. Although CCT could result in overestimated ESV by missing the smallest volume, it is not presumable that it leads to overestimated EDV [43]. It is more plausible that the overestimation using CCT is caused by the fast (<20 sec) injection of a quite large volume (2 ml/kg) of contrast medium [43]. The iodine contrast solution arrives in the right ventricle immediately after injection causing higher RV volumes by increasing preload and minimal negative inotropic effects [43–45], whereas the concentration of the contrast medium in the left ventricle was less pronounced because of dispersion in the pulmonary circulation.

The method of data acquisition and evaluation differed in the three imaging modalities. While specific sectional planes have to be defined in CMRI during the scans and cannot be changed afterwards, CCT and 3DE generate modifiable 3D volume sets with optimisation of the sectional planes later on. The subsequent reconstruction of the data sets has the advantage of being more flexible: Data sets can be reconstructed and manipulated in x, y, z axes as often as necessary for correctly fitted short-axes [46]. Consequently, the risk of oblique short-axis slices arising is higher in CMRI because image adapting cannot be repeated after the examination. The selection of oblique short-axis slices would tend to overestimate the volume calculated from summated short-axis slices [47]. To avoid overestimation and also underestimation we included the area between the tricuspid and pulmonary valve near the heart base in CMRI and CCT short-axes. If only slices were included in which the right ventricle was a unit, the volumes would be underestimated in cases of oblique slices. Otherwise, volume would be overestimated if the tricuspid and the pulmonary valves would be ignored and the right atrium and pulmonary artery would be included in the volume. However, the assessment in the short-axis orientation complicates identification of the position of the pulmonary and tricuspid valves, which could also result in erroneous measurements [24]. Moreover, partial volume effects of the basal slice inherent in the short-axis method were a reason for wrong results [48]. Consequently, the determination of the last included slice near the heart base can be a source of error in cases of CCT and CMRI measurements.

For that reason, the 4D software of 3DE has the advantage of not using the short-axis alone but also with two axial planes for contour delineation during evaluation. This could be useful because the RV outflow tract and the pulmonary valve are orientated angular to the short-axis slices, which makes determination of RV contour complicated for CCT as well as for CMRI [41]. Nevertheless, because of its thin structure the pulmonary valve was often difficult to see in the coronal plan in 3DE, which resulted in impaired contour delineation. Consequently, the measurements were not improved by this technique in our study.

For evaluation of the RV volume and function, all three modalities used physic-based algorithms integrated in their software application that make no assumptions of RV geometry. Different quantification software used for CMRI, CCT and 3DE were described as a potential source of discrepancies [23]. In our study, the software RV-Volume® (TomTec) was used to evaluate the 3DE data. In agreement with other publications, in which the same software was used, the RV volumes were underestimated [6]. However, the underestimation was not always significant [9,10]. Certainly, small inaccuracies in the software used are possible, but the main difference still exists because of the limited visualisation of the RV free wall.

The EF represents one of the most important functional parameters of the right ventricle. Impaired function results in inadequate SV and can lead to heart failure [3]. In our study we found similar results for EF and SV in CMRI and CCT values. Consequently, the function parameters are not affected by the method CMRI or CCT. A possible explanation for the significantly higher EF using 3DE is the more pronounced underestimation of the RV volume expressed as a percentage of CMRI in ESV (53% of CMRI) than in EDV (46% of CMRI). Nevertheless, the possible negative inotropic effect of prolonged anaesthesia cannot be ruled out. As expected the SV was significantly lower using 3DE than in the other methods, caused by underestimation of the RV volumes.

CMRI is the gold standard for RV quantification in human medicine. Drawbacks of this technique are the necessity for anaesthesia, the long investigation time and the high costs. In cases of contraindication for CMRI (CMRI-incompatible implants), the CCT can be used. One reason for favouring the CCT investigation is the significant shorter data acquisition time. For the CCT investigation we needed 10 minutes, in contrast to the CMRI investigation which took approximately 50 minutes. The generation of acceptable 3DE RV volume data sets lasts about 10 minutes. On the contrary, limitation factors of CCT are the exposure of x-radiation and the use of potentially nephrotoxic contrast medium.

The most non-invasive technique is the 3DE. Dogs can stay awake during examination, which is especially beneficial for dogs with heart failure because general anaesthesia is often complicated by the presence of cardiac disease [49]. It is possible that in awake dogs spatial resolution of 3DE is even worse, caused by the patient moving and panting. In this situation the single-beat quantification used in this study would be preferable because the multi-volume sample acquisition probably results in stitching artefacts. On the other hand, enlargement of the RV in dogs with right-sided heart disease demands even wider field of views so the temporal resolution has to be optimised, too. Therefore, the evaluation by 3DE in awake dogs with right-sided heart disease and differing body weight is an interesting topic for further studies.

A disadvantage of 3DE is the need for intensive and time-consuming manual border correction despite using the highest grade of sensitivity for automatic contour identification. The intra-observer variability was far better than inter-observer variability. This is caused by the difficult definition of the RV border and especially the pulmonic and tricuspid valve. Furthermore, the less good inter-observer variability is probably influenced by the use of slightly different contrast and echogenicity settings used during optimisation of the raw data sets by each observer. The intra-observer variability of lower 5% is good. Therefore, each observer could reliably identify the same borders in each repeated examination. This implies that measurements should possibly be performed by the same investigator in case of studies or repeated measurements.

### Limitations

The main limitation of the study was the low number of study participants so that for general statements and creation of reference values studies with a larger number of participants (allocated into groups for gender, age and weight) are needed. Additionally, the influence of a prolonged anaesthesia and of differences in patient positioning as well as differences of ventilation technique cannot be ruled out completely. Moreover, in our study we investigated only dogs without relevant heart failure in anaesthesia. Therefore, further studies with dogs during wakefulness and dogs with right heart disease are needed to evaluate if early changes of the RV volume and function quantified with the 3D techniques can be used as a predictor of development of right-sided heart failure in dogs in the future. Further limitations of the study design were the low number of observers for the 3DE data and the limited imaging quality of 3DE data sets, as already mentioned. Probably, technical improvements, contrast echocardiography or multi-beat analysis will achieve better spatial and temporal resolution.

## Conclusion

The excellent definition of endocardial borders and the high temporal resolution of CMRI images enable assessment of RV volume and function and confirm its use as gold standard. The CCT data sets also show well depiction but with significant overestimation of RV volumes, therefore these methods are not interchangeable. A good alternative for quantification of RV is the 3DE technique. Although 3DE values are not directly comparable with CCT and CMRI, caused by its underestimation of RV volumes, the excellent correlation let assume that 3DE is a feasible alternative.

## Endnotes

^a^VetHDO® High definition oscillometry, S + B MedVet GmbH, Germany

^b^L-Polamivet®, IntervetGmBH, Germany

^c^Diazepam-ratiopharm®, CP-Pharma GmBH, Germany

^d^Narcofol®, CP-Pharma GmBH, Germany

^e^Isofluran®, CP-Pharma GmBH, Germany

^f^Vivid E9; GE healthcare, Germany

^g^4D RV-Volume®; TomTec Imaging Systems GmbH, Germany

^h^Achieva 3.0 T; Philips, Netherlands

^i^Radio-Translucent Foam Monitoring Electrodes, Germany

^j^Sense Flex Small/Medium; Philips, Netherlands

^k^MR Systems Achieva, Philips, Netherlands

^l^Brilliance 64; Philips, Netherlands

^m^Xenetix® 350 mg Iod/ml, Guerbet GmBH, Germany

^n^Medrad Vistron CT Injection System, Medrad, Pa.

^o^Extended Brilliance Workspace, Philips, Netherlands

^p^Cardiac viewer, Philips, Netherlands

^q^LV/RV Analysis software, Philips, Netherlands

^r^SAS9.2, SAS Institute, Cary North Carolina, USA and Prism, GraphPad Software, La Jolla, USA.

## Abbreviations

3DE: Three-dimensional echocardiography
CCT: Cardiac computed tomography
CMRI: Cardiac magnetic resonance imaging
ECG: Electrocardiogram
EDV: End-diastolic volume
EF: Ejection fraction
ESV: End-systolic volume
Fps: Frames per second
RV: Right ventricular
SV: Stroke volume
SD: Standard deviation.
